# P-200. Evolving Beyond the Pandemic: A Four-Year Review of Infectious Disease Cases at the Pakistan’s First Dedicated Infectious Diseases Hospital

**DOI:** 10.1093/ofid/ofaf695.422

**Published:** 2026-01-11

**Authors:** Muneeba Ahsan Sayeed, Elisha Shalim

**Affiliations:** Sindh Infectious Diseases Hospital & Research Centre/Dow University of Health Sciences, Karachi, Sindh, Pakistan; Sindh Infectious Diseases Hospital & Research Centre/Dow University of Health Sciences, Karachi, Sindh, Pakistan

## Abstract

**Background:**

Sindh Infectious Diseases Hospital & Research Centre (SIDH & RC) is the Pakistan’s first dedicated infectious diseases facility, which was established in Karachi, Pakistan, in July 2020 to address the COVID-19 pandemic during which it, managed over 5,000 COVID-19 cases. Initially, there was a general perception that the hospital would become redundant once the pandemic ended. However, contrary to this belief, the facility evolved over time, broadening its services to manage a wide spectrum of infectious diseases beyond COVID-19.

Annual Trend of Infectious Disease Cases

**Figure:**
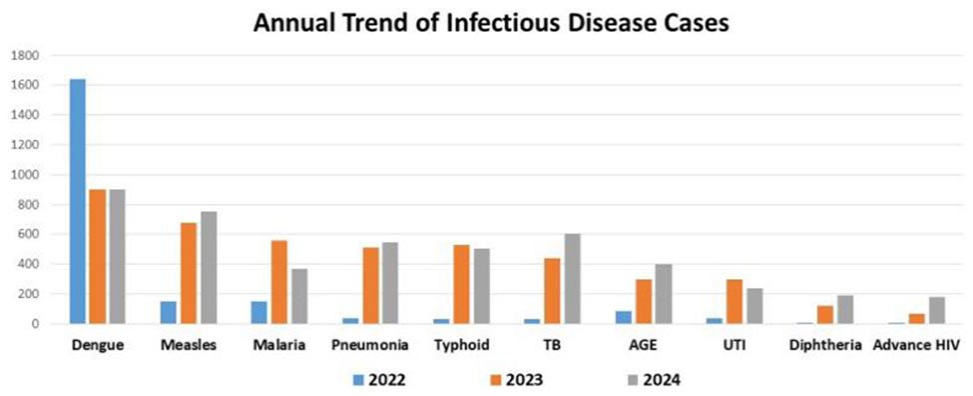

**Methods:**

A retrospective observational study was conducted to assess the trend of major infectious diseases from 2021 to 2024 at SIDH & RC, Karachi, Pakistan.

**Results:**

A total of 11,406 patients were admitted. The most commonly treated condition was dengue (n = 3,554), followed by measles (n = 1,578), malaria (n = 1,079), pneumonia (n = 1,096), typhoid (n = 1,069), tuberculosis (n = 1,080), acute gastroenteritis (n = 785), urinary tract infections (n = 572), diphtheria (n = 309), and advanced HIV (n = 261).

Distinct age-related patterns were observed . Measles and diphtheria primarily affected children, with mean ages of 5.3 and 12.0 years, respectively. Typhoid mainly impacted adolescents (mean age 17 years), while dengue and malaria predominantly affected young adults (mean ages 27–28 years). Advanced HIV had a mean patient age of 37 years, while UTIs, TB, and pneumonia occurred more frequently in older adults, with mean ages of 41, 45, and 57 years, respectively.

The highest in-hospital mortality rate was observed in diphtheria (24.6%), followed by tuberculosis (21.2%), pneumonia (20.7%), advanced HIV (19.9%), measles (6.2%), pyelonephritis (3.5%), acute gastroenteritis (3.18%), malaria (2.1%), and dengue (1.1%).

**Conclusion:**

Following the decline of COVID-19 in 2022, Pakistan faced successive outbreaks of dengue, malaria and typhoid in 2022-2023, measles in 2023, and diphtheria in 2024—largely due to floods and disrupted immunization—prompting the hospital to establish specialized units. This underscores the critical need for a dedicated infectious diseases facility to manage emerging public health threats and outbreaks.

**Disclosures:**

All Authors: No reported disclosures

